# Economic evaluation of human albumin use in patients with nephrotic syndrome in four Brazilian public hospitals: pharmacoeconomic study: Avaliação econômica do uso de albumina humana em pacientes com síndrome nefrótica em quatro hospitais públicos brasileiros: estudo de farmacoeconomia

**DOI:** 10.1590/1516-3180.2016.0048030516

**Published:** 2017-04-20

**Authors:** Leonardo Augusto Kister de Toledo, Antônio Carlos Beisl Noblat, Harrison Floriano do Nascimento, Lúcia de Araújo Costa Beisl Noblat

**Affiliations:** I MSc. Pharmacist, Hospital Universitário Professor Edgard Santos (HUPES), Universidade Federal da Bahia (UFBA), Salvador (BA), Brazil.; II MD, PhD. Nephrologist, Head of Complex Care Management Division, Hospital Universitário Professor Edgard Santos (HUPES), Universidade Federal da Bahia (UFBA), Salvador (BA), Brazil.; III MSc. Economist, Hospital Universitário Professor Edgard Santos (HUPES), Salvador (BA), Brazil.; IV Pharmacyst, PhD. Professor, School of Pharmacy, Universidade Federal da Bahia (UFBA), and Education and Research Manager, Hospital Universitário Professor Edgard Santos (HUPES), Salvador (BA), Brazil.

**Keywords:** Economics, pharmaceutical, Economics, medical, Nephrotic syndrome, Cost-benefit analysis, Albumins

## Abstract

**CONTEXT AND OBJECTIVE:**

In 2004, the Brazilian National Health Surveillance Agency (Agência Nacional de Vigilância Sanitária, ANVISA) published a resolution establishing guidelines for albumin use. Although the published data do not indicate any definitive conclusions about the benefits of albumin use in patients with nephrotic syndrome (NS), the guidelines recommend this procedure only in cases of edema that is refractory to use of diuretics. The aim here was to analyze albumin use among patients with nephrotic syndrome.

**DESIGN AND SETTING:**

Pharmacoeconomic study conducted in four large public referral hospitals for nephrology services in northeastern Brazil.

**METHOD:**

Cost-effectiveness and cost-utility economic evaluations were performed on a concurrent cohort of patients with nephrotic syndrome, who were divided into two groups according to compliance or noncompliance with the guidelines. Quality-of-life data were obtained from the SF36 and CHQ-PF50 questionnaires.

**RESULTS:**

This study enrolled 109 patients (60% adults and 56% women); 41.3% were using albumin in accordance with the guidelines. The weight, diuresis and fluid balance parameters were more cost-effective for patients who adhered to the guidelines. Regarding days of hospitalization avoided, the incremental ratio showed a daily cost of R$ 55.33, and guideline-compliant patients were hospitalized for five days or fewer. The quality of life improved by 8%, and savings of R$ 3,458.13/QALY (quality-adjusted life year) for the healthcare system were generated through guideline compliance.

**CONCLUSION:**

The economic analyses of this study demonstrated that there were greater cost benefits for patients whose treatment followed the guidelines.

## RESUMO

**CONTEXTO E OBJETIVO:**

Em 2004, a Agência Nacional de Vigilância Sanitária (ANVISA) publicou uma resolução que estabelece diretrizes para o uso de albumina. Embora os dados publicados não indiquem conclusões definitivas sobre os benefícios do uso de albumina em pacientes com síndrome nefrótica (SN), a diretriz recomenda o procedimento apenas em casos de edema refratário ao uso de diuréticos. O objetivo aqui foi analisar o uso de albumina em pacientes com síndrome nefrótica.

**TIPO DE ESTUDO E LOCAL:**

Estudo farmacoeconômico realizado em quatro grandes hospitais públicos de referência em serviços de nefrologia no nordeste do Brasil.

**MÉTODO:**

Foram realizadas avaliações econômicas do tipo custo-efetividade e custo-utilidade em uma coorte concorrente de pacientes com síndrome nefrótica, divididos em dois grupos de acordo com o cumprimento ou descumprimento das diretrizes. Dados de qualidade de vida foram obtidos a partir dos questionários SF36 e CHQ-PF50.

**RESULTADOS:**

Este estudo incluiu 109 pacientes (60% adultos e 56% mulheres); 41,3% estavam usando albumina conforme as diretrizes. Os parâmetros de peso, diurese e balanço hídrico foram mais custo-efetivos para pacientes que aderiram às diretrizes. Quanto a dias de internação evitados, a razão incremental mostrou um custo diário de R$ 55,33, sendo que os pacientes que seguiram as diretrizes ficaram cinco dias a menos internados. A qualidade de vida melhorou 8%, gerando economia de R$ 3,458.13/QALY *(quality-adjusted life year)* para o sistema de saúde por meio do cumprimento das diretrizes.

**CONCLUSÃO:**

As análises econômicas deste estudo demonstraram maiores benefícios em termos de custo para os pacientes cujo tratamento seguiu as diretrizes.

## INTRODUCTION

Healthcare needs have expanded exponentially over recent years, thus increasing the demand for more effective results. A number of analysis tools are available to public policy managers, to enable justification of decisions that are made. One of these tools is economic evaluation, which is characterized by comparative analysis of different interventions in terms of costs and their consequences. Economic evaluation has been regarded as an excellent decision-making support tool.^1^^,^^2^^,^^3^^,^^4^^,^^5^Production of clinical protocols and therapeutic guidelines rationalizes the use and safety of technologies, since they organize logical thinking towards positive results in terms of economy, effectiveness, safety and efficiency.^6^

Albumin is an endogenous liver-synthesized protein. It is present at high concentrations in human plasma and is primarily responsible for maintaining intravascular oncotic pressure.^7^^,^^8^^,^^9^^,^^10^ As a pharmaceutical product, human albumin is an injectable blood product from human plasma that is found at hyperosmotic concentrations in plasma (4% to 25%). Its primary therapeutic indications are for restoration of oncotic and iso-osmotic pressure (4%), and also, although less indicated, for plasma volume restoration.^7^^,^^10^

Because of variability in the way in which human albumin is used in Brazil, as well as its high cost, the National Health Surveillance Agency (Agência Nacional de Vigilância Sanitária, ANVISA) published guidelines for therapeutic use of this drug in 2004.^7^ Brazil’s expenditure on imported blood products in 2010 reached US$ 330 million and, within this amount, 12 tons of human albumin were imported, at a cost of approximately US$ 50 million.^11^

One of the formal indications for the use of human albumin stated within the scope of ANVISA’s guidelines is nephrotic syndrome (NS). This involves the presence of large-scale edema that is refractory to diuretics, thereby endangering these patients’ lives (due to pleural effusion, pericardial effusion or bulky ascites). In these cases, treatment with albumin would be short-term and would aim to resolve the patient’s acute decompensation. Presence of hypoalbuminemia alone in patients with NS (a condition that is caused by the disease) does not justify albumin use.^7^

NS is a clinical condition characterized by the presence of massive proteinuria, edema, hypoproteinemia and dyslipidemia. Massive proteinuria is defined as excretion of more than 3.5 g of protein per 1.73 m^2^ of body surface area within 24 hours or more than 50 mg/kg of bodyweight within 24 hours through the urinary tract. NS affects both adults and children and is primarily caused by kidney disease (idiopathic or primary NS) or by various pathological conditions (secondary NS)^12^^,^^13^^,^^14^^,^^15^ Generalized edema is the main complication of NS, since it can lead to serious conditions such as pulmonary edema, heart failure and hypertension.^13^ Formation of this edema can be explained through pathophysiological mechanisms that are activated due to decreased glomerular filtration rates resulting from prior renal disease, inadequate sodium excretion in the distal tubules and hypoalbuminemia.^13^^,^^15^^,^^16^ The treatments for nephrotic syndrome depend on two factors, namely, the patient’s general condition and the type of primary renal disease. In cases of generalized edema, the first procedure should be grounded in removing the patient from the critical state.

The use of human albumin in association with diuretics as the first choice for patients with NS has been extensively discussed in the literature.^17^ Some studies have shown that human albumin enhances the effect of diuretics,^13^^,^^18^^,^^19^^,^^20^^,^^21^ while other studies have failed to show any difference in the results from comparisons between ways of using diuretics: on a stand-alone basis or in association with albumin.^17^^,^^22^^,^^23^^,^^24^^,^^25^ Some other studies have contraindicated this combination of human albumin and diuretics for treating edema in NS,^26^ while yet others have shown benefit from stand-alone use of albumin.^27^

Knowledge of patients’ profiles and economic evaluation of human albumin use among patients with NS can be valuable tools in decision-making for the healthcare system. This information can assist managers in spreading the use of these guidelines to other healthcare units, thereby optimizing disease management and use of public resources, or it may spur a review of standards.

## OBJECTIVE

The aim of this study was to analyze albumin use among patients with nephrotic syndrome, from the perspective of the Brazilian National Health System (Sistema Único de Saúde, SUS), through a pharmacoeconomic study.

## METHODS 

### Method, population and data source

Cost-effectiveness and cost-utility economic evaluations were performed on a concurrent cohort of patients with nephrotic syndrome that was observed between December 2010 and July 2012.

This study was conducted in four public referral hospitals for nephrology services that are located in Salvador, Bahia. The criteria for selecting these institutions comprised their care profile (referral centers for nephrology within the state’s public health network), level of human albumin consumption and formal acceptance of participation.

The study population consisted of consecutive adult and pediatric patients with nephrotic syndrome, who were monitored from the start to the end of their treatment and were divided into two groups:

- Group 1: patients whose treatment followed the instructions for human albumin use, as recommended by ANVISA, i.e. human albumin was only used in cases of large-scale edema that was refractory to diuretics;
- Group 2: patients whose treatments did not follow policy guidelines.

Patients were required to agree to and sign a free and informed consent statement in order to join the study.

The research team was independent from the clinical team, and only allocated the case to one or other of the groups shortly after the beginning of the treatment prescribed. Thus, if after the case had been diagnosed as nephrotic syndrome and the use of albumin alone or albumin in association with diuretics was prescribed as the first-choice treatment, this patient would belong to group 2 (treatment not following the guidelines). If the patient was using diuretics alone and an inadequate therapeutic response was obtained (i.e. the patient was refractory to diuretics), and then albumin use was added, this patient would be allocated to group 1, since the guidelines indicate that albumin should only be used for patients with nephrotic syndrome in cases of large-scale edema that was refractory to diuretics.

In order to check the allocations of patients to groups 1 and 2 that the researchers made, all cases were reviewed and validated weekly by a nephrologist.

### Data-gathering

Clinical, laboratory, socioeconomic and demographic data were gathered upon confirmation of the case of nephrotic syndrome during hospitalization. The main sources of information used were the human albumin stock movement records in the pharmacy service, daily medical prescriptions, hospitalization censuses and medical records, along with interviews with patients and/or guardians. The data-gathering was conducted by trained field workers who had been instructed about all the study methodology and research instruments.

The variables were grouped into general data, socioeconomic and demographic data, clinical data, indications for human albumin, hospital length of stay, reason for discharge, deaths and quality-of-life data.

The quality-of-life data were obtained by applying two generic health-related quality-of-life questionnaires, which have both been adapted and validated for the Brazilian population. The Medical Outcomes Survey Short Form 36 (SF36),^28^^,^^29^ which consists of 36 questions addressing eight quality-of-life domains, was used for adults. Scale scores are converted into values from 0 (worst quality of life) to 100 (best quality of life). Adolescents aged 12 and over could also answer this questionnaire.

The “50-item, parent complete short form, Child Health Questionnaire” (CHQ-PF50)^30^ was used for pediatric patients up to the age of 12 years. This can be applied to these patients either directly or indirectly through their parents or guardians.^31^^,^^32^ It consists of ten patient-related health concepts and four family-related concepts, in order to measure the emotional impact of child health on the adult responsible for that child. The results range from 0 to 100, with higher scores indicating better quality of life.

The patients included in this study gave responses to the quality-of-life questionnaires at two stages: before the onset of treatment and four weeks after the first application.

The Short Form 6D (SF-6D) algorithm was applied to data gathered through the SF36. This enabled direct measurement of utility, through calculation of quality-adjusted life years (QALYs).^33^

Cost data were obtained by calculating the patients’ direct treatment costs during the hospitalization period through the absorption costing technique, since the participating hospitals did not have systematic computerized overhead cost information to enable their estimation. The direct costs used in this study were drawn from the medical billing sectors of the institutions and were gathered from the patients’ billing sheets (patient/days of hospitalization), in accordance with the SUS procedures table. Costs were adjusted for inflation to June 2013, based on the Market General Price Index (IGP-M) of the Getúlio Vargas Foundation (Fundação Getúlio Vargas, FGV). The study was conducted from the economic perspective of SUS.

All the cases were presented and discussed before officially defining their inclusion in the study, at meetings in which both the data-gatherers and the advisory members of the project (two pharmacists, one economist and one nephrologist) participated. All data collected were checked by a supervisor before database entry. Where divergences and/or lack of data were noted, the questionnaires were reevaluated by the researchers using complementary information and were analyzed again by the supervisor.

### Data analysis

The data were entered into the Microsoft Excel 2007 software and were analyzed using SPSS version 20.0. Continuous variables were expressed as means and standard deviations. The Kolmogorov-Smirnov test was used to evaluate the normality pattern of the distribution of continuous variables. P-values of less than 0.05 with 95% confidence interval were considered to be statistically significant. Categorical variables were reported as proportions and were tested using the chi-square test.

The cost-effectiveness economic analysis was performed starting from cost-effectiveness mean ratios and, when necessary, incremental cost-effectiveness ratio (ICER) analysis was applied, in which costs and health outcomes were calculated by dividing the difference in strategy cost by the difference in health results for each group. For the cost-effectiveness economic analysis, the effectiveness indicators used to compare the groups were: weight, diuresis, water balance and days of hospitalization avoided.

The mean cost-utility ratio was also used for cost-utility analysis and, when required, the incremental cost-utility ratio (ICUR) was applied. In this case, the health outcome measurements were scores from the results of the quality-of-life questionnaires, transformed into quality-adjusted life years (QALYs).

Univariate sensitivity analysis was performed. Each parameter was assessed separately within its range of variation, while the other remained constant. The objective was to ascertain the influence of the parameter analyzed on the final result, so as to determine whether or not this was sensitive to change.

The study was approved by the Research Ethics Committee of the Prof. Edgard Santos University Hospital Complex, Federal University of Bahia, under report number 063/2007.

## RESULTS

One hundred and nine patients were enrolled by the end of the study period, and the treatments for 45 (41.3%) of them followed the human albumin use guidelines. Two patients were excluded because of lack of information for the economic analysis that had been planned. 

In Group 1, 64% (29/45) were pediatric patients with a mean age of 16 years, and the CHQ-PF50 questionnaire was applied in most cases. In group 2, 77% (49/64) were adult patients with a mean age of 28 years, and the SF36 was used in most cases. Comparison between the average ages of the groups showed differences (P < 0.0001). Table 1 shows the comparison groups according to the four hospitals surveyed.

**Table 1:**
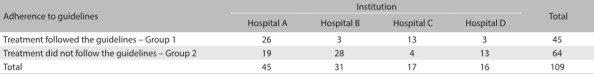
Total number of patients with nephrotic syndrome in each study group, according to hospital of origin, in Salvador, Bahia, in 2012

The demographic and socioeconomic data are shown in Table 2. Overall, an average of four people were living with each patient, and this average was also maintained in the group analysis.

**Table 2:**
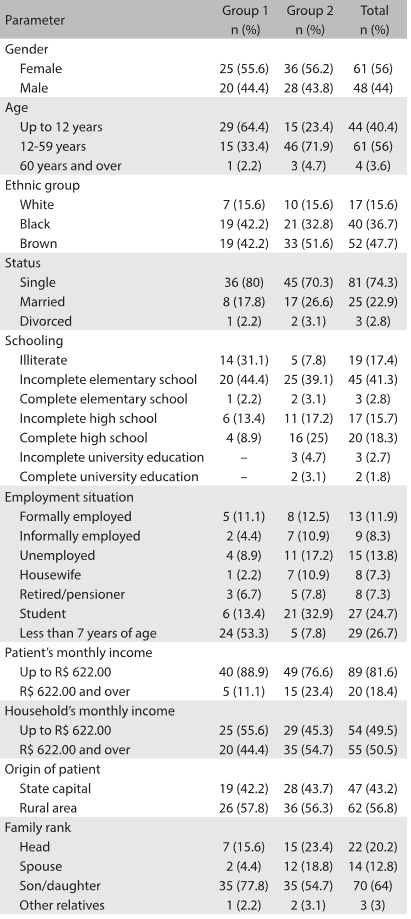
General characteristics of patients with nephrotic syndrome treated at four hospitals in Salvador, Bahia, in 2012, according to group

In Group 1, 77.8% (35/45) of the patients were not in their first treatment, whereas a balance was noted in Group 2 between first and non-first treatment, with 50% (32/64) of the cases in each treatment situation.

The overall average hospitalization cost for SUS (Sistema Único de Saúde, the Brazilian public health system) in this study was R$ 2,360.00, with an average of 22 days of hospitalization. The average cost for Group 1 was R$ 2,221.68, with an average of 20 days of hospitalization and it was R$ 2,498.33 for Group 2, with an average of 25 days of hospitalization. The incremental cost-effectiveness ratios for days of hospitalizations and urine output parameters are shown in Table 3. The weight loss and fluid balance parameters were better in Group 1.

**Table 3:**
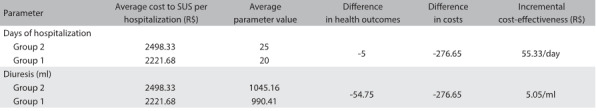
Incremental cost-effectiveness ratio according to “days of hospitalization” and “diuresis” among patients with nephrotic syndrome treated at four hospitals in Salvador, Bahia, in 2012

SUS = Sistema Único de Saúde, the Brazilian National Health System.

**Figure 1:**
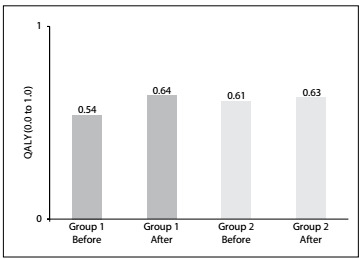
Comparison of QALY (quality-adjusted life year) indices before and after treatment with human albumin, according to groups of patients with nephrotic syndrome treated at four hospitals in Salvador, Bahia, in 2012.

Data from the quality-of-life analyses are shown in Figure 1, through QALY indices before and after the procedure for each group. Table 4 displays the ICUR.

Univariate sensitivity analyses were performed based on the ICER and ICUR results. The ICER sensitivity data showed that there was an increase of 60% in the value of avoided hospitalization days, with a difference of 2 days of hospitalization between the groups; the amount of expenditure avoided per day was only R$ 138.33. The ICUR sensitivity analysis showed that there was a 30% improvement in the quality of life, which generated savings of R$ 1,024.63 for SUS.

**Table 4:**
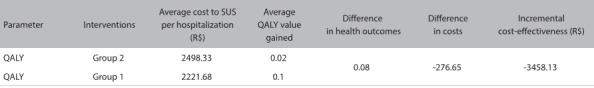
Incremental cost-effectiveness ratio per QALY of patients with nephrotic syndrome treated at four hospitals in Salvador, Bahia, in 2012

QALY = quality-adjusted life year; SUS = Sistema Único de Saúde, the Brazilian National Health System.

## DISCUSSION

This was the first pharmacoeconomic study in Brazil examining the use of human albumin among patients with nephrotic syndrome. The general economic analyses showed that the treatments that followed ANVISA’s guidelines were more cost-effective. These data suggest that these guidelines should be implemented in SUS services where they are currently not being used. This can be justified since they produce clinical and economic results for the system.

These data show that treatments have better cost-effectiveness and usefulness for patients when the guidelines are followed, even though this issue remains undefined in the literature. This seems to lead to a broad discussion on the appropriateness of using an initial association of human albumin and diuretics as the first choice in treatments for NS. It has been suggested in the literature that the clinical relevance of the effects of furosemide and albumin is still obscure and that there is no accumulated scientific evidence justifying use of this combination in the initial intervention, or as a routine treatment.^25^ The review by Elwell et al.^25^ confirms that the results from different studies are conflicting with regard to using human albumin coupled with furosemide in cases of NS. Therefore, this combination should be reserved for specific groups of patients whose doses of diuretics have been maximized or for cases of severe hypoalbuminemia.^17^

The findings from the review by Caraceni et al.^34^ show that use of human albumin alone for correcting hypoalbuminemia is not backed by scientific evidence. This confirms the stance taken in the guidelines, i.e. that thorough analysis is required before using human albumin in a direct association and as the first-choice treatment.^7^^,^^24^^,^^34^

The possible positive impact within clinical practice from using a combination of human albumin and furosemide among patients with edema is not associated with correction of hypovolemia. There is agreement that one possible explanation for the boosting of the effect of furosemide is that greater availability of the drug at its site of action is achieved.^13^^,^^35^

The present study based on economic analyses had the aim of assessing ANVISA’s guidelines regarding the use of human albumin among patients with NS. It also poses the question of whether the use of protocols and guidelines in clinical practice is relevant. It has been suggested in the literature that institutionalization and dissemination of guidelines or protocols in itself does not necessarily translate into changes in practices or into lasting clinical results.^21^ Data from one study have shown that it takes five to ten years to achieve significant results from changes in practice after implementation of these instruments.^36^

The process of establishing therapeutic guidelines should also be followed by continuing education, review of processes and monitoring of practices. These actions can definitely bring about real and positive results.^10^^,^^36^^,^^37^

Lastly, the benefits of guidelines and clinical protocols and their negative aspects and the obstacles to implementing them have been widely assessed in the literature. The information from the present study, in which the treatments for most of the cases did not follow the policy guidelines, reinforces the previously published data, thus reaffirming that merely disseminating a guideline or protocol does not warrant safer, more effective and more efficient practices for patients, government and society itself.

The assessment of the numbers of cases distributed per hospital showed the situation of bed availability within these SUS hospitals in Bahia. One of them is the largest state hospital in the public network, with approximately 1,792 beds and emergency care, whereas the others are traditional referral centers for nephrology.

Ethnic group analysis showed that brown and black individuals together accounted for more than 90% of the cases, which reflects the state’s population composition.^38^

An analysis on the patients’ origins revealed that most of them were from municipalities in rural areas of the state. This may demonstrate the fragility or lack of hospitals in rural macroregions to serve the needs of this profile of patients.

Regarding the economic analysis, the cost-effectiveness analysis results calculated from the average ratios for weight loss and fluid balance outcomes showed better relationships for Group 1. An incremental ratio method was used for the parameter of days of hospitalization, since treatments that followed the guidelines were more effective but also more costly. This allowed the hospitalization to be five days shorter but with an increased daily cost (R$ 111.08/day versus R$ 99.93/day). Thus, the ICER result for the number of days of hospitalization showed that an additional cost of R$ 55.33 will be required per day avoided. This data needs to be analyzed in the light of the reality of the lack of public hospital beds in Brazil and the consequent long waiting lists for care and attempts to make overall savings for SUS through deinstitutionalization of patients.

In fact, the additional cost should be analyzed as an investment in the system, since having a bed available five days earlier brings more benefits. Data gathered by the Organization for Economic Cooperation and Development (OECD) show that the number of beds per capita varies greatly between countries and over time.^39^ Brazil has one of the lowest levels in the Americas (1.7 beds per 1,000 population).^39^

Several other risks are associated with longer hospital stays. Hospital infection and various adverse events can be highlighted among these risks, and these give rise to additional diagnostic investigations and tests, along with greater routine stay expenses (accommodation, meals, general nursing, etc.), which all contribute towards increased hospitalization costs. These explanations ultimately indicate that investments in deinstitutionalization need to be made. Univariate sensitivity analysis showed that a difference of only two days of hospitalization between the groups would generate a 60% increase in the amount saved through avoidance of hospital stay. Thus, investment of these amounts can be justified, given the other costs of keeping patients hospitalized.

The average cost-effectiveness rate for the diuresis parameter in Group 1 was less effective and lower, in relation to the possible cost-effectiveness responses. The ICER analysis for diuresis highlighted the need to invest resources, so as to achieve clinical results better than those that have been found so far. However, in clinical practice, these shortfalls probably would not have an impact on patients great enough to justify investment.

The results from the cost-utility analysis showed that Group 1 achieved greater improvement of quality of life than Group 2. This benefit can be put back into society and into SUS.

There was an increase in quality of life of 10% for Group 1 and 2% for Group 2. For treatments that followed the guidelines, ICUR showed that there were savings of R$ 3,458.13 per QALY for SUS.

Univariate sensitivity analysis on ICUR, setting the QALY gained in Group 2 at 2%, showed that the savings for the system were maintained even when the difference in QALY gained by the groups was more than 30%. In other words, savings for SUS were generated through treatments that followed the guidelines, even with better quality of life in Group 1.

It is important to highlight that there is no high-quality evidence supporting use of albumin for treating nephrotic syndrome. Caution is still needed in using albumin, since this is not considered at all for other clinical situations. For example, a non-concurrent historical cohort study that was carried out in Brazil based on DATASUS records found that use of albumin among patients with major burns was associated with considerably increased mortality (seven times higher than when crystalloid solutions were used).^40^

The clinical evaluation on the effectiveness of intermediate outcomes for weight, diuresis and fluid balance parameters more directly related to the care process showed differences between the groups in this study. However, within clinical practice, these differences would probably not demonstrate observable relevance. Nonetheless, what can be highlighted is the difference in the number of days of hospitalization, i.e. the number of days avoided and the increased quality of life for patients whose treatments followed the guidelines. These analyses showed both clinical benefits for patients and savings for the system.

## CONCLUSION

The cost-effectiveness results, and especially the cost-utility results of this study provided information on the use of human albumin among patients with nephrotic syndrome. This information, along with other data, can guide healthcare practices within SUS from both the clinical and the economic and financial viewpoints.

Economic analyses suggest that the use of guidelines for using human albumin among patients with NS has been beneficial, since there was a gain in effectiveness and quality of life for patients and economic gains for SUS.
